# Toxic element levels in ingredients and commercial pet foods

**DOI:** 10.1038/s41598-021-00467-4

**Published:** 2021-10-25

**Authors:** Rafael Vessecchi Amorim Zafalon, Raquel Silveira Pedreira, Thiago Henrique Annibale Vendramini, Mariana Fragoso Rentas, Vivian Pedrinelli, Roberta Bueno Aires Rodrigues, Larissa Wunsche Risolia, Mariana Pamplona Perini, Andressa Rodrigues Amaral, Júlio Cesar de Carvalho Balieiro, Cristiana Fonseca Ferreira Pontieri, Marcio Antonio Brunetto

**Affiliations:** 1 School of Veterinary Medicine and Animal Science, University of São Paulo, 225, Duque de Caxias Norte Avenue, Pirassununga, São Paulo, 13635 900 Brazil; 2 Nutritional Development Center of Premier Pet, Dourado, Brazil

**Keywords:** Diseases, Risk factors

## Abstract

Nowadays, there is a growing concern about contamination of toxic metals (TM) in pet food due to the great potential for health risks of these elements. TM concentrations in commercial pet foods (n = 100) as well as in ingredients used in their composition (n = 100) were analyzed and compared to the Food and Drug Administration (FDA) maximum tolerable level (MTL), and the TM concentrations found in the different sources of carbohydrate, protein, and fat were compared. The TM concentrations were determined by inductively coupled plasma with optical emission spectrometry (ICP-OES). Concentrations above the MTL for aluminum, mercury, lead, uranium, and vanadium were observed in both dog and cat foods, and the percentage of dog foods that exceeded the MTL of these TM were: 31.9%; 100%; 80.55%; 95.83%; and 75%, respectively, and in cat foods: 10.71%; 100%; 32.14%; 85.71%; 28.57%, respectively. The MTL values of these TMs and the mean values in dog foods (mg/kg dry matter basis) (MTL [mean ± standard deviation]) were: aluminum: 200 (269.17 ± 393.74); mercury: 0.27 (2.51 ± 1.31); lead: 10 (12.55 ± 4.30); uranium: 10 (76.82 ± 28.09); vanadium: 1 (1.35 ± 0.69), while in cat foods were: aluminum: 200 (135.51 ± 143.95); mercury: 0.27 (3.47 ± 4.31); lead: 10 (9.13 ± 5.42); uranium: 10 (49.83 ± 29.18); vanadium: 1 (0.81 ± 0.77). Dry foods presented higher concentrations of most TM (P < 0.05) than wet foods (P < 0.05). Among the carbohydrate sources, there were the highest levels of all TM except cobalt, mercury, and nickel in wheat bran (P < 0.05), while among the protein sources, in general, animal by-products had higher TM concentrations than plant-based ingredients. Pork fat had higher concentrations of arsenic, mercury, and antimony than fish oil and poultry fat. It was concluded that the pet foods evaluated in this study presented high concentrations of the following TM: aluminum, mercury, lead, uranium, and vanadium.

## Introduction

Toxic metals contamination is a current concern worldwide due to its toxicity, intrinsic persistence, non-biodegradable nature, and cumulative characteristics^1,2^. It is known that toxic metals occur naturally in the environment, but their presence is mainly due to anthropic action, such as agricultural activity, which includes the application of fertilizers and soil amendments; industrial activity, related to mining and metal smelting; and burning of fossil fuels, as well as the incineration of residues and products containing toxic metals^3–6^. Therefore, industrialization, urbanization, population growth, and agriculture are causes of pollution by toxic metals worldwide^2,7^.

The soil is the most important environmental compartment and functions as a deposit for these elements, and can suffer contamination through several sources such as agricultural fertilizers; soil correctives; agrochemicals; irrigation water, sewage sludge, and other waste; atmospheric deposition of industrial sources; and urban emissions^8^. This contamination can reach areas of agricultural production and farm animals, which can have their products and by-products used in the manufacture of pet foods. In the literature, there are several studies conducted in different countries that have found toxic metal contamination with a wide range of concentrations in various types of food intended for humans such as fish, rice, vegetables, dried fruits, meat, quail meat, and bovine tissues^9–20^. Therefore, plant-based ingredients used by the pet food industry grown in contaminated soil, such as corn, rice, wheat, peas, barley, and sorghum, can accumulate toxic metals and contribute to pet food contamination. In the case of food animals, they can accumulate toxic metals when consuming contaminated foods due to soil contamination thus contaminating animal by-products, such as poultry by-products meal and meat meal, which can also contribute to pet food contamination. There is also contamination of rivers and oceans, affecting aquatic organisms that can also be used as raw material by the pet food industry. Therefore, there is great concern about the occurrence of toxic metals in pet food.

Several studies reported the toxic metals presence in canine samples of blood^21,22^, hair^21,23–26^, kidneys^27,28^, bone tissue^29^, and liver^23,27,28^ and hair samples from cats^24^. It is known that ingestion through food is the main contamination route^30–33^, therefore, it is believed that the food consumed by these animals may be contaminated by these elements, which can imply health risks for dogs and cats.

According to Instituto Pet Brasil^34^, the domiciled dog and cat population in Brazil was estimated at 54.2 and 23.9 million, respectively. According to the Brazilian Institute of Geography and Statistics (IBGE)^35^, 44.3% of the 65 million households have at least one dog and 17.7% at least one cat. The increase of the population of domiciled dogs and cats, as well as the intensification of the relationship between owners and their pets, is reflected in the expansion of the pet market in general, including the food segment. According to data from the Brazilian Association of the Pet Products Industry (ABINPET)^36^, the Brazilian pet market turnover in 2018 was R$ (Brazilian real) 22.3 billion (US$ 6.16 billion), which places Brazil as the third largest in the world, representing more than 4.7% of the global turnover in this segment. The pet food market represents 73.3% of this revenue, and it was the segment of the pet market that grew the most between 2018 and 2019 (8.4%), with a production of around 2.85 million tons^36^.

The concern about the safety of commercial dog and cat food is growing, since the food recall that occurred in the U.S. due to melamine contamination, which resulted in the death of thousands of dogs from acute kidney failure^37,38^. Recently, the pet food industry has diversified the variety of its products, with different types of ingredients, such as additives to reduce fecal odor and alternative sources of protein and carbohydrate, like vegan or grain-free diets; and processing methods, such as wet foods, which may include cheaper ingredients of lower quality and can result in the introduction of several contaminants, such as pesticides, mycotoxins, and toxic metals^38,39^.

To date, some studies have evaluated the presence of toxic metals in commercial pet foods^38,40–46^ and ingredients used by the pet food industry^47–49^. From the published data, the high aluminum concentrations in commercial pet foods observed in the studies by Fernandes^38^, da Costa^45^, and Paulelli^46^ stand out, in which aluminum concentrations up to 11,900 mg/kg food were observed, which correspond to 59.5 times the maximum limit established for these species. In small animals, the possible adverse effects associated with consuming this amount of aluminum are unclear. It is also worth highlighting the lead concentrations found in the study by Duran^40^, in which concentrations above the established limit for dogs and cats were observed. These results are considered worrying, as an excessive lead consumption has been associated with gastrointestinal signs^50,51^, neurological disorders^50,52^, damage to the hematopoietic system^53,54^, and kidney injuries^52^.

Given the relevance of the topic and the little information in the literature about toxic metal contamination in pet foods, and especially in ingredients, the goals of this study were: to evaluate the concentrations of toxic metals [aluminum (Al), antimony (Sb), arsenic (As), barium (Ba), beryllium (Be), boron (B), cadmium (Cd), chromium (Cr), cobalt (Co), iron (Fe), lead (Pb), mercury (Hg), nickel (Ni), selenium (Se), tin (Sn), uranium (U), and vanadium (V)] in ingredients used in pet food formulation and in commercial pet foods available in the Brazilian market; to compare the results with the maximum tolerated level values recommended for dogs and cats; to investigate whether there is a difference between the toxic metals’ concentrations in dry and wet foods and between the different categories (economical, premium, and super premium); to analyze if there is a difference between toxic metals concentrations in dog and cat foods and to evaluate if there is a difference between protein sources and carbohydrate sources.

## Material and methods

### Acquisition of samples

A total of 100 complete and balanced commercial pet foods for adult animals from 29 different manufacturers were purchased from pet shops located in cities of the State of São Paulo (São Carlos, Araraquara, and Indaiatuba) during the period from 09/03/2018 to 01/25/2019. The number of samples of each food type is shown in Table 1. The ingredient samples were supplied by the company Premier Pet, during the period from 09/05/2018 to 04/25/2019, and each sample of each ingredient is from a different supplier. A total of 500 g of sample of each ingredient were collected, taken from different points of the load. The ingredients analyzed in this study are listed in Table 2. The dry foods were classified into the following categories: standard, premium, and super premium. This classification was carried out based on information declared by the manufacturer on the labels of the commercial products, which is a commercial classification. The standard products are formulated with the lowest cost, with lower concentrations of nutrients such as protein and fat generally, with values close to the minimum recommendations. Premium foods, in turn, have a better selection of ingredients and higher nutrient concentrations. Finally, the super premium foods have high-quality ingredients in their formulation, providing a more adequate nutrition and may even incorporate functional ingredients.

**Table 1:** Number of samples of each type of commercial pet food analyzed.

Commercial pet foods	No. of samples
**Dog foods**	72
Standard dry foods	18
Premium dry foods	23
Super premium dry foods	20
Wet foods	11
**Cat foods**	28
Standard dry foods	4
Premium dry foods	5
Super premium dry foods	5
Wet foods	14
Total	100

**Table 2:** Analyzed ingredients and number of samples per ingredient.

Ingredients	No. of samples
**Animal by-products**	54
Chicken by-products meal	16
Beef meal	8
Fish meal	6
Feather meal	6
Fish oil	6
Pork fat	6
Poultry fat	6
**Plant-based ingredients**	40
Broken rice	6
Whole corn	10
Wheat bran	6
Soybean meal	6
Corn gluten meal 21	6
Corn gluten meal 60	6
Mineral supplement	6
Total	100

### Dry matter analysis

The samples of each dry food were ground in a Willye knife mill (Marconi MA340, Piracicaba, Brazil) (sieve of 1 mm). The wet food samples were previously dehydrated in a forced circulation oven at 55 °C for 72 h^55^, and later were ground in an analytical mill (Ika, A11 Basic Mill, Staufen, Germany). The samples of broken rice, whole corn, soybean meal, and wheat bran had to be milled in a micro-knife mill (Marconi MA048, Piracicaba, Brazil) (sieve of 1 mm).

After grinding, a sub-sample was taken for dry matter (DM) analysis in an oven at 105 °C^56^. These analyses were performed in duplicate at the Multiuser Laboratory of Animal Nutrition and Bromatology of the Department of Nutrition and Animal Production of the School of Veterinary Medicine and Animal Science of University of Sao Paulo, Pirassununga-Brazil. Repetition was performed when the variation coefficient between samples was greater than 5.0%.

### Sample preparation for the toxic metal’s determination

The preparation of all samples (except samples of fat sources) was carried out by the wet method according to Pedrinelli^57^. For all samples, 0.5 g were weighed and placed in polypropylene tubes, and afterward, 1.5 mL of HNO_3_ P.A. (65% m/v) (brand: Synth^®^) (Diadema, Brazil) and 2.0 mL of H_2_O_2_ (30% m/v) (brand: Dinâmica^®^) (Indaiatuba, Brazil) were added to each tube. After 30 min, the volume was completed with 4.5 mL of ultrapure water type I (18.2 MΩ cm resistivity; conductivity: 0.054 µS/cm; TOC: < 5 PPB [< 5 µg/L]), obtained from a Milli-Q purification system (Millipore, USA). Then, the tubes were placed in a microwave oven (Multiwave GO, Anton Paar, Austria) and were subjected to heating in two phases: in the first, the samples were heated for 20 min until reaching 180 °C in 400 W; in the second phase, the samples were heated by 180 °C at 800 W for 10 min and, subsequently, cooling was performed for 10 min. After the digestion procedure, the samples were transferred to polyethylene tubes and ultrapure water type I (18.2 MΩ cm resistivity; conductivity: 0.054 µS/cm; TOC: < 5 PPB [< 5 µg/L]), obtained from a Milli-Q purification system (Millipore, USA), was added until they reached 25 mL of volume. Blank solutions were subjected to the same procedure to verify the quality of the reagents. The procedure was performed in duplicate. The samples’ preparation by microwave digestion was carried out in the Laboratory of Biorigin Brazil (Lençóis Paulista, Brazil).

The preparation of the fat samples was performed using the methodology adapted from Llorent-Martínez^58^. Acid digestion of samples was carried out in a microwave SW-4 model Speed Wave (Berghof, Germany). An aliquot of approximately 0.15 g of sample was weighed directly into the digestion vessel, then 5 mL of diluted HNO_3_ (25% HNO_3_ and 75% ultrapure water) was added to each oil sample. The HNO_3_ used was P.A. (65% m/v) (brand: Synth^®^) (Diadema, Brazil), and ultrapure water used was a type I (18.2 MΩ cm resistivity; conductivity: 0.054 µS/cm; TOC: < 5 PPB [< 5 µg/L]), obtained from a Milli-Q purification system (Millipore, USA). Subsequently, the digestion procedure was performed in four stages of heating. First, the samples were heated for 5 min until reaching 90 °C at 700 W. In the second stage, the temperature was maintained at 90 °C for 3 min at 600 W. In the third stage, the temperature was increased for 10 min to 170 °C at 600 W. In the last stage, the temperature was kept at 170 °C for another 7 min at 600 W. After this stage, the samples were cooled and ultrapure water was added until the volume reached 15 mL. Two blank solutions were included for every 18 fat samples. The digestion of fat samples was carried out at the analytical center of the chemistry institute of the State University of Campinas (Campinas, Brazil).

### Determination of elements in ICP-OES

The determination of aluminum (Al), antimony (Sb), arsenic (As), barium (Ba), beryllium (Be), boron (B), cadmium (Cd), chromium (Cr), cobalt (Co), iron (Fe), lead (Pb), mercury (Hg), nickel (Ni), selenium (Se), tin (Sn), uranium (U), and vanadium (V) was performed by optical emission spectrometry with inductively coupled plasma [ICP-OES (ICPE-9000, Shimadzu of Brazil, Barueri, Brazil)], at the Multiuser Laboratory of Animal Nutrition and Bromatology of the Department of Nutrition and Animal Production of the School of Veterinary Medicine and Animal Science of the University of Sao Paulo, Pirassununga—Brazil.

For As, Hg, Sb, and Se determination a hydride generator (hydride ICP, Elemental Scientific, Omaha, USA) coupled to the ICP-OES was used. To avoid cross-contamination, ultrapure water type I (18.2 MΩ cm resistivity; conductivity: 0.054 µS/cm; TOC: < 5 PPB [< 5 µg/L]), obtained from a Milli-Q purification system (Millipore, USA) was used between the samples to clean the system and, for every five analyses of samples determined by the ICP-OES, the system was cleaned with 1.0 g/100 mL nitric acid P.A. (65% m/v) (brand: Synth^®^) (Diadema, Brazil). The calibration curves were prepared using multi-element solutions with a certificate of analysis and traceability to NIST (National Institute of Standards and Technology, Gaithersburg, MD, USA) of 100 mg/L for the elements Al, As, B, Ba, Be, Cd, Co, Cr, Fe, Hg, Ni, Pb, Sb, Se, Sn, and V, and monoelementary solutions (with a certificate of analysis and traceability to NIST) of 100 mg/L of U. The reference material used was SpecSol^®^ (Jacareí, Brazil) and was purchased from the company Quimlab (www.quimlab.com.br). The metal concentrations of the reference material are traced to the following NIST standards: Al: NIST 928; As: NIST 3103a; B: NIST 3107; Ba: NIST 3104a; Be: NIST 3105a; Cd: NIST 928; Co: NIST 928; Cr: NIST 3112a; Fe: NIST 928; Hg: NIST 3133; Ni; NIST 928; Pb: NIST 928; Se: NIST 3149; Sb: NIST 136f; Sn: NIST 3161a; U: NIST 3164; V: NIST 3165. The curves were prepared with the aid of automatic pipettes and falcon tubes were used. Calibration curves were prepared one day before analysis. For As, Sb, Se, and Hg the calibration curves had the following points: 0.001—0.05—0.1—0.5—1—2 ppm. For the other metals analyzed the calibration curves had the following points: 0.001—0.05—0.1—0.5—1—2—5—10 ppm.

The emission line wavelengths of each element were: As—193,759; Sb—206,833; Se—196,090; Hg—184,950; A1—167,081; B—249,773; Ba—455,403; Be—234,861; Cd—226.502; Co—238,892; Cr—205.552; Fe—238,204; Ni—231,604; Pb—220,353; Sn—189,989; U—263,553; and V—292,402. Operational conditions are presented in Table 3.

**Table 3:** Operational conditions of inductively coupled plasma optical emission spectrometry (ICP-OES) with axial configuration.

Parameter	Characteristics
Radiofrequency power (kW)	1.2
Plasma gas flow rate (L/min)	10
Auxiliary gas flow rate (L/min)	0.6
Sample uptake rate (s)	30
Nebulizer gas flow rate (L/min)	0.7
Nebulizer type	Concentric
Spray chamber	Cyclone
Replicates	2

### Statistical analysis

For comparisons between dry and wet food, dog and cat food, categories of dry foods, carbohydrate sources, and protein sources, the Shapiro–Wilk test was performed to assess the normality of the residues and the F test to verify the homogeneity of the variances. For data that did not show a normal distribution, the generalized linear mixed model with a logarithmic link function was used to stabilize the residues. After data transformation, ANOVA was performed and, when there was a difference between the groups, the Tukey test was performed. The analyses described above were performed using the Statistical Analysis System (SAS) software version 9.4 (SAS Institute, North Carolina, USA) and p values below 0.05 were considered significant.

The results found in commercial foods were compared with the MTLs established by the FDA^59^ in mg/kg of dry matter, descriptively. For the elements whose values were not indicated by FDA^59^ (B, Ba, and Sn), the MTL values of the most sensitive mammal to each of the elements were used, according to the Mineral Tolerance of Animals^60^ and, for iron, the legal limit values of European Union legislation were used, according to regulation 1831/2003/EC, expressed in FEDIAF^61^, also in mg/kg of dry matter.

For the elements that showed concentrations above the MTL of FDA^59^ or legal limit expressed in FEDIAF^61^, to better investigate whether the values observed in food are a real health issue, it was calculated how much animals would consume of these elements per kg of body weight (BW) per day if they ate these analyzed foods. Thus, a simulation was carried out with dogs of different sizes (5 kg, 15 kg, 30 kg, and 50 kg) and, in the case of cats, simulations were carried out with variation in body weight between 3 and 5 kg. Simulations were performed with animals of different sizes, as the daily amounts consumed of the metals were calculated per kg of BW, not per kg of metabolic weight. Thus, the amount consumed per kg of BW differs, so that the smaller animals consume greater amounts when compared to larger animals.

The maintenance energy requirement for dogs was calculated using the equation 95 kcal × BW^0.75^, and for cats, through the equation 100 kcal × BW^0.67^. To calculate the daily amount of food to be provided, the metabolizable energy (ME) values declared on the labels of the analyzed foods were used and, for foods that did not contain this information stated on the label, the ME was estimated using the method described by the NRC^62^.

### Ethical approval

The experimental protocol was conducted according to ethical principles in human and animal experimentation and was approved by the Commission on Ethics in the Use of Animals of the School of Veterinary Medicine and Animal Science of the University of Sao Paulo (protocol number 6717110219).

## Results

### Toxic metals concentrations in commercial pet foods

Through ICP-OES methodology, it was possible to determine 17 elements. In dog foods, the elements Sb, Ba, Cr, Sn, Fe, and Hg were detected in all samples (Table 4). In cat foods, the metals Cr, Hg, Sb, Fe, and Sn were detected in all samples analyzed (Table 5). In both dog and cat foods, the elements As, B, Be, and Se were below the detection limit of 0.05 mg/kg in all samples (Tables 4 and 5). Regarding the comparisons of the results with the MTLs by FDA^59^, in dog foods, values above the MTL were observed for the following elements: Al, Pb, Co, Hg, U, and V. In cat foods, values above MTL were observed for Al, Cr, Hg, Pb, U, and V. In addition, one food had Fe concentration above the legal limit.

**Table 4:** Detected toxic metal concentrations (mg/kg DM) in the 72 dog foods evaluated and comparison with the maximum tolerated level established by the FDA (2011) and/or legal limit expressed by FEDIAF (2020).

Toxic metals	MTL	LL	Mean ± SD	Minimum–maximum	% above MTL (n)	% of samples with detection (n)
Aluminum (Al)	200^a^	–	269.17 ± 393.74	0–2406	31.9 (23)	98.6 (71)
Antimony (Sb)	40^b^	–	2.10 ± 0.48	1.56–4.90	0 (0)	100 (72)
Arsenic (As)	12.5^b^	–	–	–	0 (0)	0 (0)
Barium (Ba)	100^a^	–	27.12 ± 21.17	0.38–96.41	0 (0)	100 (72)
Beryllium (Be)	5^b^	–	–	–	0 (0)	0 (0)
Boron (B)	150^a^	–	–	–	0 (0)	0 (0)
Cadmium (Cd)	10^b^	–	2.92 ± 1.77	0–6.86	0 (0)	88.9 (64)
Lead (Pb)	10^b^	–	12.55 ± 4.30	0–21.82	80.55 (58)	97.22 (70)
Cobalt (Co)	2.5^b^	–	1.65 ± 2.36	0–14.11	6.94 (5)	91.67 (66)
Chromium (Cr)	10^b^	–	4.73 ± 1.20	0.90–7.74	0 (0)	100 (72)
Tin (Sn)	100^a^	–	9.88 ± 1.58	6.63–14.62	0 (0)	100 (72)
Iron (Fe)	–	1420	338.64 ± 213.40	18.56–1367.32	0 (0)	100 (72)
Mercury (Hg)	0.27^b^	–	2.51 ± 1.31	1.11–7.72	100 (72)	100 (72)
Nickel (Ni)	50^b^	–	1.67 ± 0.80	0–3.48	0 (0)	93.05 (67)
Selenium (Se)	–	0.568	–	–	0 (0)	0 (0)
Uranium (U)	10^b^	–	76.82 ± 28.09	0–122.05	95.83 (69)	95.83 (69)
Vanadium (V)	1^b^	–	1.35 ± 0.69	0–3.94	75 (54)	93.05 (67)

*MTL* maximum tolerated level, *LL* FEDIAF (2020) legal limit, *SD* standard deviation.

^a^Reference value of the most sensitive mammal according to the National Research Council (2005).

^b^Reference value according to the United States Food and Drug Administration (2011).

**Table 5:** Detected toxic metals concentrations (mg/kg DM) in the 28 cat foods evaluated and comparison with the maximum tolerated level of the FDA (2011) and/or legal limit expressed by FEDIAF (2020).

Toxic metals	MTL	LL	Mean ± SD	Minimum–maximum	% above MTL (n)	% of samples with detection (n)
Aluminum (Al)	200^a^	–	135.51 ± 143.95	0–582.10	10.71 (3)	96.43 (27)
Antimony (Sb)	40^b^	–	2.38 ± 1.21	1.68–6.08	0 (0)	100 (28)
Arsenic (As)	12.5^b^	–	–	–	0 (0)	0 (0)
Barium (Ba)	100^a^	–	10.80 ± 10.68	0—30.15	0 (0)	96.43 (27)
Beryllium (Be)	5^b^	–	–	–	0 (0)	0 (0)
Boron (B)	150^a^	–	–	–	0 (0)	0 (0)
Cadmium (Cd)	10^b^	–	2.18 ± 1.39	0–6.34	0 (0)	89.29 (25)
Lead (Pb)	10^b^	–	9.13 ± 5.42	0–25.75	32.14 (9)	92.86 (26)
Cobalt (Co)	2.5^b^	–	0.66 ± 0.69	0–2.49	0 (0)	82.14 (23)
Chromium (Cr)	10^b^	–	3.90 ± 1.75	0.87–10.22	3.57 (1)	100 (28)
Tin (Sn)	100^a^	–	9.56 ± 2.18	3.96–13.98	0 (0)	100 (28)
Iron (Fe)	–	1420	383.05 ± 344.87	21.39–1871.16	3.57 (1)	100 (28)
Mercury (Hg)	0.27^b^	–	3.47 ± 4.31	1.20–18.87	100 (28)	100 (28)
Nickel (Ni)	50^b^	–	1.13 ± 1.33	0–4.94	0 (0)	78.57 (22)
Selenium (Se)	–	0.568	–	–	0(0)	0(0)
Uranium (U)	10^b^	–	49.83 ± 29.18	0–111.94	85.71 (24)	96.43 (27)
Vanadium (V)	1^b^	–	0.81 ± 0.77	0–2.87	28.57 (8)	78.57 (22)

*MTL* maximum tolerated level, *LL* FEDIAF (2020) legal limit, *SD* standard deviation.

^a^Reference value of the most sensitive mammal according to the National Research Council (2005).

^b^Reference value according to the United States Food and Drug Administration (2011).

**Figure 1:**
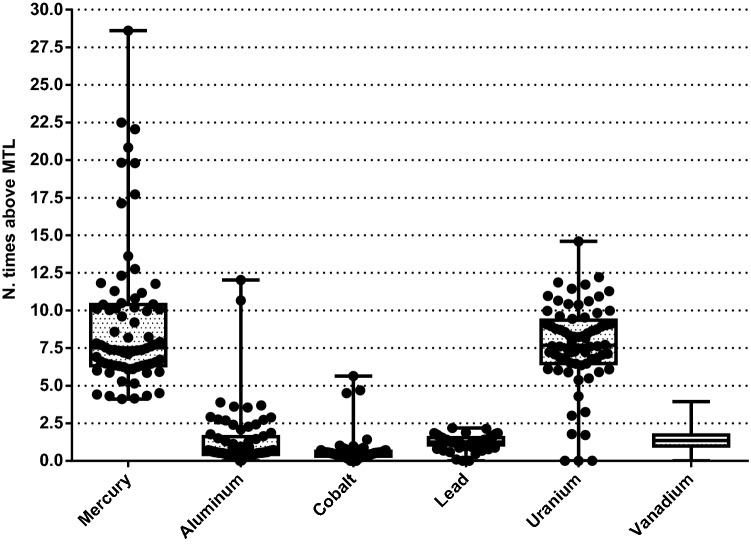
Number of times above the maximum tolerated level (MTL) for toxic metals that had values above that limit, in commercial dog foods.

**Figure 2:**
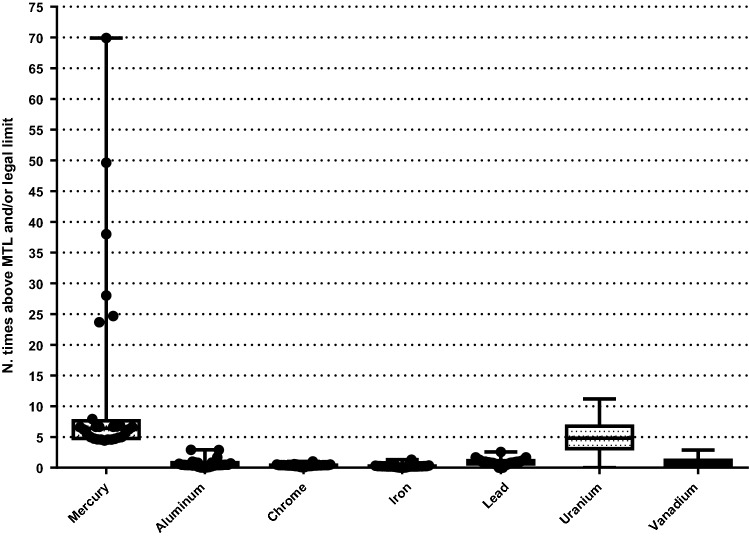
Number of times above maximum tolerated level (MTL) and/or legal limit for toxic metals that had values above that limit, in commercial cat foods.

**Figure 3:**
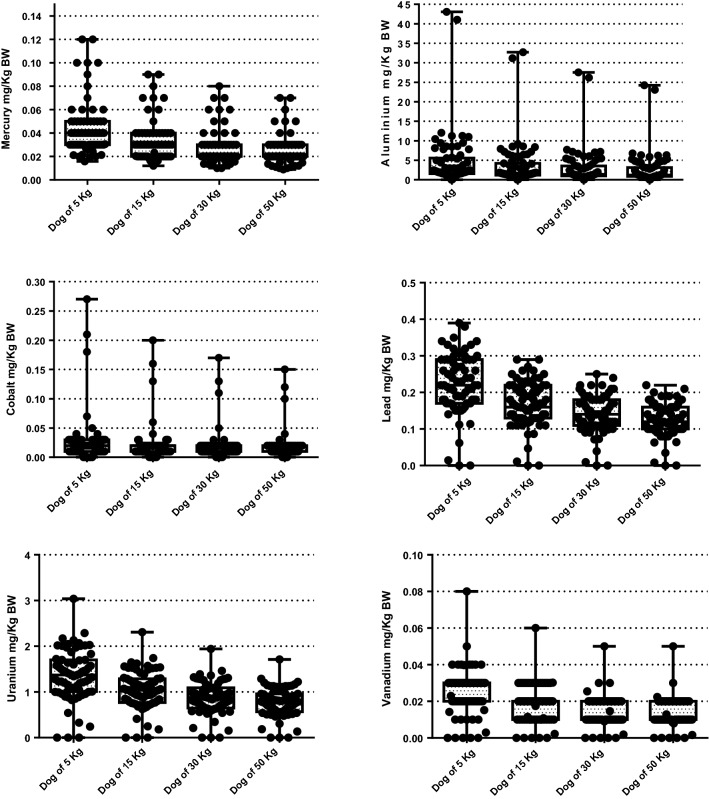
Estimated intake of the toxic metal that exceeded the maximum tolerated level (MTL) per kg of body weight (BW) of dogs of different sizes.

**Figure 4:**
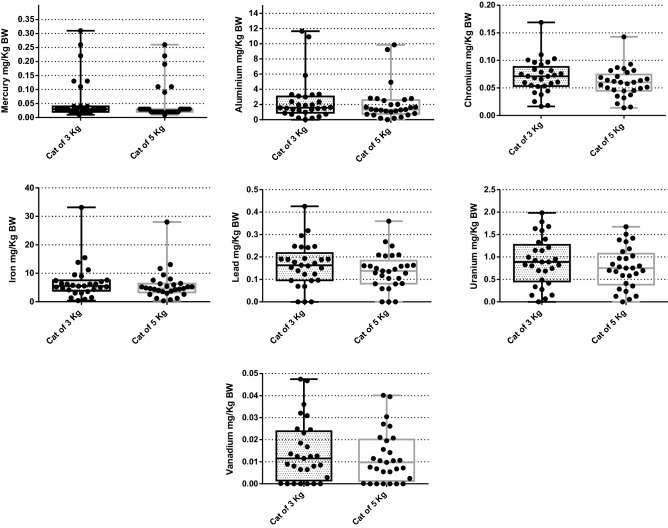
Estimated intake of toxic metals that exceeded the maximum tolerated level (MTL) and/or legal limit per kg of body weight (BW) of cats of two different sizes.

The number of times that dog and cat foods exceeded the MTL are shown in Figs. 1 and 2, respectively. The percentage of dog foods that exceeded the MTL of TMs were: 31.9% (Al); 100% (Hg); 80.55% (Pb); 95.83% (U); and 75% (V), respectively, and in cat foods: 10.71% (Al); 100% (Hg); 32.14% (Pb); 85.71% (U); 28.57% (V), respectively. Figures 3 and 4 show the quantity (in mg) that the analyzed foods provide per kg of BW of each TM that presented values above the MTL. In the comparison between dry and wet foods, higher concentrations of Al (P < 0.0001), Ba (P < 0.0001), Cd (P = 0.0008), Co (P < 0.0001), Cr (P = 0.0043), Hg (P < 0.0001), Ni (P = 0.0014), Pb (P < 0.0001), U (P < 0.0001), and V (P = 0.0011) in dry foods were observed, while wet foods had higher Fe concentrations (P < 0.0001) (Table 6).

**Table 6:** Comparison between the detected toxic metals concentrations (mg/kg) in dry and wet foods in mg/kg of dry matter.

Toxic metals	Dry foods	SEM	Wet foods	SEM	*P*
Aluminum (Al)	272.95^a^	1.9077	108.14^b^	2.0798	< 0.0001
Antimony (Sb)	2.3034^a^	0.1752	1.7988^a^	0.2682	0.1429
Barium (Ba)	26.1969^a^	0.591	11.6018^b^	0.6812	< 0.0001
Cadmium (Cd)	3.0493^a^	0.2016	1.7146^b^	0.2619	0.0008
Lead (Pb)	13.3327^a^	0.4216	6.3767^b^	0.505	< 0.0001
Cobalt (Co)	1.6788^a^	0.1496	0.4394^b^	0.1326	< 0.0001
Chromium (Cr)	4.8554^a^	0.2544	3.4159^b^	0.3696	0.0043
Tin (Sn)	9.9174^a^	0.3636	9.4197^a^	0.6138	0.4927
Iron (Fe)	323.04^b^	2.0754	435.19^a^	4.1723	< 0.0001
Mercury (Hg)	3.2469^a^	0.2081	1.3552^b^	0.2328	< 0.0001
Nickel (Ni)	1.758^a^	0.1531	0.8002^b^	0.1789	0.0014
Uranium (U)	80.9642^a^	1.039	34.1501^b^	1.1688	< 0.0001
Vanadium (V)	1.423^a^	0.1377	0.5407^b^	0.1471	0.0011

*SEM* standard error mean, *P* probability of significance.

^a,b^Means followed by different letters in the lines differed (P < 0.05).

As for comparisons between dog and cat foods, higher concentrations of Al (P = 0.0033), Ba (P = 0.0116), and Fe (P < 0.0001) in wet dog foods were observed, compared to wet cat foods, which presented higher U concentrations (P = 0.0331) (Table 7). Dry dog foods had higher concentrations of Al (P < 0.0001), Ba (P < 0.0001), Co (P = 0.0104), and U (P < 0.0001), than dry cat foods, which presented higher concentrations of Fe (P < 0.0001) and Hg (P < 0.0001) (Table 8). Regarding the comparisons between the different dog food categories, the standard category had the highest concentrations of Ba (P < 0.0001), Fe (P < 0.0001), and U (P < 0.0001), while the super-premium category showed the highest concentrations of Al (P < 0.0001) (Table 9). As for this same comparison in cat foods, the premium category showed the highest levels of U (P = 0.0084), and the super premium category presented the highest concentrations of Fe (P < 0.0001). Regarding Hg, the premium category showed higher concentrations than the super premium category (P = 0.0359), and the standard category did not differ from the others (Table 10).

**Table 7:** Comparison of detected toxic metals concentrations (mg/kg) between wet foods for dogs and cats.

Toxic metals	Wet dog foods	SEM	Wet cat foods	SEM	*P*
Aluminum (Al)	115.84^a^	3.2452	102.08^b^	2.7003	0.0033
Antimony (Sb)	1.7858^a^	0.4029	1.8091^a^	0.3595	0.9659
Barium (Ba)	13.718^a^	1.1167	9.9391^b^	0.8426	0.0116
Cadmium (Cd)	1.7299^a^	0.3966	1.7026^a^	0.3487	0.9591
Lead (Pb)	5.9801^a^	0.7373	6.6883^a^	0.6912	0.4936
Cobalt (Co)	0.3862^a^	0.1874	0.4813^a^	0.1854	0.7255
Chromium (Cr)	3.4801^a^	0.5625	3.3654^a^	0.4903	0.8789
Tin (Sn)	9.7042^a^	0.9393	9.1961^a^	0.8105	0.685
Iron (Fe)	460.81^a^	6.4724	415.07^b^	5.445	< 0.0001
Mercury (Hg)	1.3323^a^	0.348	1.3732^a^	0.3132	0.9314
Nickel (Ni)	0.6435^a^	0.2419	0.9233^a^	0.2568	0.4479
Uranium (U)	31.1574^b^	1.683	36.5015^a^	1.6147	0.0331
Vanadium (V)	0.5088^a^	0.2151	0.5658^a^	0.201	0.8493

*SEM* standard error mean, *P* probability of significance.

^a,b^Means followed by different letters in the lines differed (P < 0.05).

**Table 8:** Comparison of detected toxic metals concentrations (mg/kg) between dry foods for dogs and cats.

Toxic metals	Dry dog foods	SEM	Dry cat foods	SEM	*P*
Aluminum (Al)	296.8200^a^	2.2059	168.9400^b^	3.4738	< 0.0001
Antimony (Sb)	2.1563^a^	0.1880	2.9443^a^	0.4586	0.0852
Barium (Ba)	29.5342^a^	0.6958	11.6557^b^	0.9124	< 0.0001
Cadmium (Cd)	3.1375^a^	0.2268	2.6646^a^	0.4363	0.3643
Lead (Pb)	13.7379^a^	0.4746	11.5672^b^	0.9090	0.0488
Cobalt (Co)	1.8725^a^	0.1752	0.8350^b^	0.2442	0.0104
Chromium (Cr)	4.9510^a^	0.2849	4.4387^a^	0.5631	0.4354
Tin (Sn)	9.9140^a^	0.4031	9.9319^a^	0.8423	0.9847
Iron (Fe)	316.61^b^	2.2782	351.0200^a^	5.0073	< 0.0001
Mercury (Hg)	2.7167^b^	0.2110	5.5569^a^	0.6300	< 0.0001
Nickel (Ni)	1.8538^a^	0.1743	1.3406^a^	0.3094	0.1975
Uranium (U)	85.0520^a^	1.1808	63.1533^b^	2.1239	< 0.0001
Vanadium (V)	1.5062^a^	0.1571	1.0605 ^a^	0.2752	0.2137

*SEM* standard error mean, *P* probability of significance.

^a,b^Means followed by different letters in the lines differed (P < 0.05).

**Table 9:** Comparison of detected toxic metals concentrations (mg/kg) between different categories of dry dog foods.

Toxic metals	Standard	SEM	Premium	SEM	Super premium	SEM	*P*
Aluminum (Al)	282.79^b^	3.9637	276.62^b^	3.468	332.67^a^	4.0784	< 0.0001
Antimony (Sb)	2.0298^a^	0.3358	2.3084^a^	0.3168	2.0952^a^	0.3237	0.8135
Barium (Ba)	45.7217^a^	1.5938	23.0179^b^	1.0004	22.4592^b^	1.0597	< 0.0001
Boron (B)	0.000000153^a^	0.000092	0.000000153^a^	0.000082	0.000000153^a^	0.000087	1
Cadmium (Cd)	3.5462^a^	0.4567	3.1519^a^	0.3702	2.9306^a^	0.3828	0.5783
Lead (Pb)	14.8503^a^	0.9083	13.5389^a^	0.7672	12.9657^a^	0.8052	0.2868
Cobalt (Co)	2.1111^a^	0.3425	1.7649^a^	0.277	1.7816^a^	0.2985	0.6803
Chromium (Cr)	5.2126^a^	0.5381	4.901^a^	0.4616	4.7731^a^	0.4885	0.8242
Tin (Sn)	9.738^a^	0.7355	9.676^a^	0.6486	10.3461^a^	0.7192	0.7553
Iron (Fe)	337.6^a^	4.3308	296.17^c^	3.5884	321.24^b^	4.0077	< 0.0001
Mercury (Hg)	2.4559^a^	0.3694	2.9791^a^	0.3599	2.6497^a^	0.364	0.5902
Nickel (Ni)	2.0304^a^	0.3359	1.8084^a^	0.2804	1.7472^a^	0.2956	0.799
Uranium (U)	97.1009^a^	2.3226	83.8141^b^	1.909	75.6316^c^	1.9446	< 0.0001
Vanadium (V)	1.6406^a^	0.3019	1.5607^a^	0.2605	1.3226^a^	0.2572	0.7039

*SEM* standard error mean, *P* probability of significance.

^a,b^Means followed by different letters in the lines differed (P < 0.05).

**Table 10:** Comparison of detected toxic metals concentrations (mg/kg) between different categories of dry cat foods.

Toxic metals	Standard	SEM	Premium	SEM	Super premium	SEM	*P*
Aluminum (Al)	180.54^a^	6.7182	204.63^a^	6.3973	123.97^b^	4.9794	< 0.0001
Antimony (Sb)	2.9313^a^	0.856	3.8247^a^	0.8746	2.0742^a^	0.6441	0.319
Barium (Ba)	9.2112^a^	1.5175	13.6626^a^	1.653	11.6045^a^	1.5234	0.1997
Boron (B)	0.0000004158^a^	0.000322	0.0000004158^a^	0.000288	0.0000004158^a^	0.000288	1
Cadmium (Cd)	2.006^a^	0.7082	3.4741^a^	0.8336	2.3821^a^	0.6902	0.4003
Lead (Pb)	9.0741^a^	1.5062	15.1531^a^	1.7409	9.9758^a^	1.4125	0.0412
Cobalt (Co)	0.4962^a^	0.3522	1.2259^a^	0.4952	0.7151^a^	0.3782	0.5
Chromium (Cr)	3.6575^a^	0.9562	5.5653^a^	1.055	3.9369^a^	0.8873	0.3614
Tin (Sn)	8.5373^a^	1.4609	10.8585^a^	1.4737	10.1211^a^	1.4228	0.5585
Iron (Fe)	274.59^c^	8.2854	354.18^b^	8.4164	409.02^a^	9.0446	< 0.0001
Mercury (Hg)	6.8257^ab^	1.3063	7.1737^a^	1.1978	2.9249^b^	0.7648	0.0359
Nickel (Ni)	0.9347^a^	0.4834	2.0178^a^	0.6353	0.988^a^	0.4445	0.318
Uranium (U)	57.7126^b^	3.7984	74.2822^a^	3.8544	56.3769^b^	3.3579	0.0084
Vanadium (V)	0.7046^a^	0.4197	1.5588^a^	0.5584	0.8469^a^	0.4116	0.4369

*SEM* standard error mean, *P* probability of significance.

^a,b^Means followed by different letters in the lines differed (P < 0.05).

### Toxic metals concentrations in ingredients

**Figure 5:**
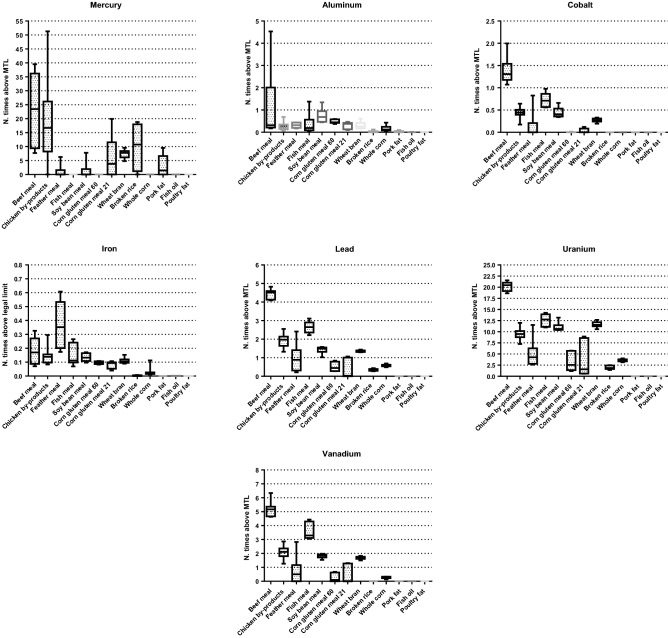
Number of times above maximum tolerated level (MTL) and/or legal limit for toxic metals in ingredients of animal and vegetable origin.

Regarding the comparisons between ingredients used as a carbohydrate source, wheat bran showed higher concentrations of Al, Ba, Cr, Fe, Pb, Sn, and U, compared to whole corn and broken rice (P < 0.05) (Table 11). Whole corn showed the highest Ni concentrations (P = 0.0007). There was no difference between carbohydrate sources for the other elements analyzed (P > 0.05) (Table 12). As for comparisons between protein sources, there was a difference between the ingredients for most of the elements analyzed (P < 0.05), except for Co (P > 0.05) (Table 12). Regarding fat sources, only Al, As, Cd, Fe, Hg, Ni, Sb, and Sn were detected in samples of at least one type of ingredient. Pork fat had higher concentrations of As, Hg, and Sb compared to fish oil and poultry fat. There was no difference between fat sources in the concentrations of the other detected TMs (Table 13). In Fig. 5, the number of times that each type of ingredient exceeded the MTL for the elements found in concentrations above this limit in commercial pet foods is illustrated. The results of the toxic metal concentrations in the analyzed mineral supplements are shown in Table 14. The sample of dicalcium phosphate analyzed was the one that presented the largest number of elements above the detection limit, so that only Se and As were in concentrations below the detection limit.

**Table 11:** Detected toxic metals concentrations (mg/kg) in the evaluated carbohydrate sources.

Toxic metals	Wheat bran	SEM	Whole corn	SEM	Broken rice	SEM	*P*
Aluminum (Al)	61.1269^a^	3.1918	33.5225^b^	1.8309	4.8543^c^	0.8995	< 0.0001
Barium (Ba)	21.8277^a^	1.9073	0.3219^b^	0.1794	0.3189^b^	0.2305	< 0.0001
Lead (Pb)	13.5606^a^	1.5034	5.8264^b^	0.7633	3.5085^b^	0.7647	< 0.0001
Cobalt (Co)	0.6809^a^	0.3369	4.158E − 07^a^	0.000204	4.158E − 07^a^	0.000263	0.9993
Chromium (Cr)	2.5956^a^	0.6577	0.4819^b^	0.2314	–	–	0.0084
Tin (Sn)	7.2633^a^	1.1002	3.4455^b^	0.587	1.9679^b^	0.5727	0.001
Iron (Fe)	156.06^a^	5.1	40.9944^b^	2.0247	4.2234^c^	0.839	< 0.0001
Mercury (Hg)	2.00^a^	0.5774	–	–	3.2166^a^	0.8021	0.2443
Nickel (Ni)	1.7282^b^	0.5367	5.3118^a^	0.7288	0.1796^b^	0.173	0.0007
Uranium (U)	116.38^a^	4.4042	35.6404^b^	1.8879	19.5955^c^	1.8072	< 0.0001
Vanadium (V)	1.6922^a^	0.5311	0.2646^b^	0.1627	–	–	0.0177

*SEM* standard error mean, *P* probability of significance.

^a,b^Means followed by different letters in the lines differed (P < 0.05).

**Table 12:** Detected toxic metals concentrations (mg/kg) in the evaluated protein sources.

Toxic metals	Beef meal	SEM	CGM 21	SEM	CGM 60	SEM	Fish meal	SEM	Feather meal	SEM	Soybean meal	SEM	Chicken by-products meal	SEM	*P*
Al	221.85^a^	5.2660	62.0251^d^	3.2152	95.4710^c^	3.9890	73.6629^d^	3.5039	62.8442^d^	3.2364	148.43^b^	4.9737	49.1379^e^	1.7525	< 0.0001
As	0.0000^a^	< 0.0001	0.2392^a^	0.1997	0.0000^a^	0.0002	0.000^a^	0.0002	0.000^a^	0.0002	0.000^a^	0.0002	0.000^a^	0.0001	1.000
B	–	–	–	–	–	–	–	–	–	–	20.213	1.8354	–	–	–
Ba	136.59^a^	4.1321	0.2957^d^	0.2220	0.3342^d^	0.2360	3.7413^c^	0.7897	4.4303^c^	0.8593	9.9167^b^	1.2856	11.8609^b^	0.8610	< 0.0001
Cd	6.0219^a^	0.8676	–	–	–	–	3.9481^ab^	0.8112	–	–	–	–	2.6884^b^	0.4099	0.0027
Co	3.4913^a^	0.6606	0.0807^ab^	0.1159	0.0063^ab^	0.0325	1.8144^ab^	0.5499	0.3442^b^	0.2395	1.1074^ab^	0.4296	1.1094^b^	0.2633	0.0006
Cr	10.0963^a^	1.1234	0.7348^d^	0.3499	1.0127^d^	0.4108	5.8104^ab^	0.9841	1.7835^cd^	0.5452	2.4648^bcd^	0.6409	3.7841^bc^	0.4863	< 0.0001
Fe	257.08^b^	5.6688	115.86^e^	4.3943	136.41^d^	4.7682	213.07^c^	5.9592	522.37^a^	9.3307	191.20^c^	5.6451	199.02^c^	3.5269	< 0.0001
Hg	6.195^a^	0.880	1.632^b^	5.222	–	–	–	–	0.284^b^	0.217	0.350^b^	0.242	5.045^a^	0.5615	< 0.0001
Ni	4.1005^a^	0.7159	0.2195^b^	0.1913	0.0553^ab^	0.0960	2.3281^ab^	0.6229	0.5534^b^	0.3037	2.0148^ab^	0.5795	1.4225^b^	0.2982	0.0001
Pb	44.2657^a^	2.3523	3.5215f.	0.7661	5.1178^ef^	0.9236	26.4557^b^	2.0998	9.6875^de^	1.2707	14.2407^cd^	1.5406	19.0577^c^	1.0914	< 0.0001
Sb	0.3732^b^	0.216	10.76^a^	1.6406	–	–	–	–	8.72^a^	2.0884	–	–	0.1691^b^	0.1679	< 0.0001
Sn	22.8075^a^	1.6885	6.4901^d^	1.0400	6.5571^d^	1.0454	15.1641^b^	1.5898	8.7319^cd^	1.2064	8.3889^cd^	1.1824	12.7898^bc^	0.8941	< 0.0001
U	201.68^a^	5.0209	36.3262^e^	2.4606	31.9928^e^	2.3091	126.17^b^	4.5856	50.154^d^	2.8912	110.28^b^	4.2871	94.6199^c^	2.4318	< 0.0001
V	5.1708^a^	0.8040	0.4283^c^	0.2672	0.2473^c^	0.2030	3.5753^ab^	0.7719	0.7391^bc^	0.351	1.8167^abc^	0.5503	2.1038^bc^	0.3626	< 0.0001

*Al* aluminum, *As* arsenic, *B* boron, *Ba* barium, *Cd* cadmium, *Co* cobalt, *Cr* chromium, *Cu* copper, *Fe* iron, *Hg* mercury, *Ni* nickel, *Pb* lead, *Sb* antimony, *Sn* tin, *U* uranium, *V* vanadium, *Zn* zinc, *CGM 21* corn gluten meal 21, *CGM 60* corn gluten meal 60, *SEM* standard error mean, *P* probability of significance.

^a,b^Means followed by different letters in the lines differed (P < 0.05).

**Table 13:** Detected toxic metals concentrations (mg/kg) in the evaluated fat sources.

Toxic metals	Pork fat	Fish oil	Poultry fat	SEM	P
Mercury (Hg)	0.81^a^	0.00^b^	0.00^b^	0.249	0.0546
Arsenic (As)	1.01^a^	0.34^b^	0.00^b^	0.158	0.0013
Antimony (Sb)	0.99^a^	0.12^b^	0.00^b^	0.185	0.0033
Aluminum (Al)	4.51	0.51	0.01	1.976	0.2433
Cadmium (Cd)	0.73	0.54	0.52	0.352	0.8978
Iron (Fe)	0.00	1.15	0.37	0.444	0.2045
Nickel (Ni)	0.15	0.00	0.28	0.177	0.5461
Tin (Sn)	7.12	10.38	6.85	1.752	0.3131

*SEM* standard error mean, *P* probability of significance.

^a,b^Means followed by different letters in the lines differed (P < 0.05) by tukey test.

**Table 14:** Toxic metals concentrations (mg/kg) of in the analyzed mineral supplements.

Toxic metals	Sodium chloride 1	Sodium chloride 2	Potassium chloride 1	Potassium chloride 2	Calcium carbonate	Dicalcium phosphate
Aluminum (Al)	11.85	< 0.05	< 0.05	< 0.05	316.39	499.54
Antimony (Sb)	< 0.05	< 0.05	< 0.05	< 0.05	< 0.05	0.64
Arsenic (As)	< 0.05	< 0.05	< 0.05	< 0.05	< 0.05	< 0.05
Barium (Ba)	0.27	0.27	0.19	0.46	1.46	1.16
Beryllium (Be)	< 0.05	< 0.05	< 0.05	< 0.05	< 0.05	6.37
Boron (B)	< 0.05	< 0.05	< 0.05	< 0.05	< 0.05	< 0.05
Cadmium (Cd)	< 0.05	< 0.05	< 0.05	< 0.05	5.83	10.12
Lead (Pb)	3.85	2.62	0.72	3.49	66.29	92.51
Cobalt (Co)	< 0.05	< 0.05	< 0.05	< 0.05	4.44	3.05
Chromium (Cr)	< 0.05	< 0.05	< 0.05	< 0.05	13.41	24.21
Tin (Sn)	0.50	0.86	0.12	0.06	25.71	29.65
Iron (Fe)	6.55	2.89	7.55	2.20	140.62	10,792.48
Mercury (Hg)	< 0.05	< 0.05	< 0.05	< 0.05	6.08	4.63
Nickel (Ni)	< 0.05	< 0.05	< 0.05	< 0.05	8.44	45.84
Selenium (Se)	< 0.05	< 0.05	< 0.05	< 0.05	< 0.05	< 0.05
Uranium (U)	13.95	12.60	10.31	15.81	281.24	673.24
Vanadium (V)	0.26	0.02	< 0.05	0.07	7.83	29.246

*SEM* standard error mean, *P* probability of significance.

^a,b^Means followed by different letters in the lines differed (P < 0.05).

## Discussion

Of all the toxic metals analyzed, it is noteworthy that selenium and iron are considered essential, as they have known biological functions important for the maintenance of vital functions, therefore they must be present in the food so that only the excess is considered harmful. The main findings of this study regarding contamination by toxic metals in commercial pet foods were the high concentrations of Al, Pb, Hg, U, and V.

### Aluminum

Concerning aluminum, in dog and cat foods, 31.9% and 10.71% had levels above the MTL, respectively. However, more than 75% of the analyzed foods exceeded the MTL less than 2.5 times. In a study conducted by da Costa^45^, which evaluated commercial foods for dogs and cats available in the Brazilian market, Al concentrations also determined by ICP-OES were above MTL in several pet foods. Fernandes^38^ also observed high Al concentrations (determined by instrumental neutron activation) in commercial dog foods, which ranged from 58 to 11900 mg/kg food, which corresponds to 59.5 times the MTL. In a study conducted by Paulelli^46^, Al concentrations in commercial dog and cat foods ranged from 12 to 519 mg/kg food, and dog foods had higher Al concentrations than cat foods, which is consistent with the present findings.

It is worth mentioning that there is little information in the literature regarding the Al toxicity in small animals, so the maximum intake that these animals can tolerate is not known. The MTL used in the present study as a parameter was extrapolated from the species most sensitive to this element, however, dogs and cats may be more tolerant to Al intake, consequently, a smaller proportion of the analyzed foods would be exceeding the aluminum tolerance for these species.

It is known that the Al absorption in the gastrointestinal tract is low (less than 1.0% in animals) and can be influenced by some factors such as solubility, pH, and chemical presentation^63^. In addition, it has been shown that the presence of citric acid in the diet results in increased Al absorption in humans^64^, as well as increased Al retention in tissues of laboratory animals^65–68^. This inclusion of citric acid could increase the absorption of aluminum present in food by the gastrointestinal tract of dogs and cats and, especially in foods with high concentrations of this element, it would increase the risk of adverse effects.

In a study conducted by Katz^69^, four groups of Beagle dogs received sodium aluminum phosphate, in dosages of 0.0%, 0.3%, 1.0%, and 3.0% for 6 months. Complete blood count, biochemical analyses, and urine tests were performed, in which no changes suggestive of toxicity were observed. The authors concluded that the “No Observed Adverse Effect Level” (NOAEL) was 70 mg/kg BW/day. Pettersen^70^ evaluated the effects of consuming high Al doses (0–1143 mg/kg BW for males; 0–1251 mg/kg BW for females) in Beagles. Toxicity was limited to an acute and transient decrease in food consumption and a concomitant decrease in body weight, observed only in males. There were no changes in serum biochemistry, variables obtained from blood counts, and urinalysis. In the present study, it was calculated how much animals would consume of Al per kg of BW per day if they were fed these diets, and it was observed that none of the analyzed foods provided the amount of 70 mg/kg BW/day. This suggests that the Al concentrations observed in this study do not imply risks of intoxication. Based on data from the present study, no food provided more than 45 mg/kg BW/day.

Regarding the Al concentrations in the analyzed ingredients, higher concentrations were found in the wheat bran, however, none of the samples of this ingredient exceeded the MTL for this element. The beef meal was the protein source with the highest Al concentrations, and 25% of the samples exceeded more than twice the MTL.

### Mercury

As for Hg concentrations, values much higher than the MTL were observed in all foods analyzed, both for dogs and cats. Other studies have evaluated Hg levels in commercial pet foods^38,42–44,64^, but none of them observed concentrations as high as in the present study. In contrast, in another study conducted by our research group, Pedrinelli^57^ found high Hg levels in homemade foods for dogs and cats, in which 70.7% and 76.0% of dog and cat foods exceeded MTL, respectively. In a study conducted by Luippold^42^, the authors evaluated the presence of mercury in 101 commercial foods (dry and wet foods) for dogs and cats, all of which contained fish as the main ingredient. The values found ranged from 0.001 to 0.604 mg/kg with an average concentration of 0.043 mg/kg, so some foods exceeded the MTL (0.27 mg/kg), but less than three times at most.

The Hg MTL recommended by FDA^59^ was established based on the study by Charbonneau^71^ with cats, in which different oral doses of methylmercury, the most toxic form of Hg, were tested and it was concluded that 0.02 mg Hg/kg BW/day did not cause adverse effects after 2 years. Thus, the FDA^59^ estimated that for a dog or cat to consume this daily amount, the food must have a Hg content of 0.27 mg/kg dry matter, which was established as the MTL for Hg. Most foods in the present study, both for dogs and cats, provided amounts of mercury above 0.02 mg/kg BW/day. Among the adverse effects of high Hg intake, Charbonneau^71^ observed ataxia, loss of balance, and motor incoordination in the group of cats that consumed 0.176 mg of Hg/kg BW/day after 14 weeks, and these same signs were observed after 40 weeks in the group that consumed 0.074 mg of Hg/kg BW/day. Histopathological findings demonstrated neurological injuries.

It should be noted that the Hg MTL was determined based on the methylmercury consumption, the most toxic form of Hg found mainly in aquatic organisms. In the present study, the methodology employed did not allow quantifying the different Hg forms present in the samples, only the total Hg concentrations. It is assumed, however, that methylmercury is not the predominant form in the analyzed foods, since this organometallic compound is found in fish and other aquatic organisms, in which over 90% of the Hg is in the form of methylmercury^72,73^. In most of the analyzed foods, fish by-products or other aquatic organisms were not present, according to what was stated on the labels. In addition, none of the samples of fish meal and fish oil analyzed in our study had Hg concentrations above the detection limit. This suggests that the chemical Hg form in the analyzed foods is not methylmercury, i.e., it may be a less toxic form, which in the concentrations found in this study, may not have adverse effects on the health of pets. However, this aspect needs to be further investigated.

In our study, dry foods had higher Hg concentrations than wet foods, results opposite to those observed by Luippold^42^ and Paulelli^46^. Concerning dry foods, those intended for cats had higher Hg concentrations than those found in dog food. This could be attributed to the greater inclusion of protein to meet the higher protein requirement of the feline species, since animal protein sources, more specifically beef meal and chicken by-products, were the ingredients with the highest Hg contamination and are widely used in dry pet food formulation.

As previously mentioned, no fish meal samples had Hg concentrations above the detection limit, although fish is considered the main Hg poisoning pathway in humans^74,75^. In the study conducted by Kim^44^, when determining toxic metals in commercial dry dog foods, the authors found higher Hg concentrations in food whose primary protein source was based on fish by-products, compared to those foods whose main protein source was by-products derived from red meat and poultry. A variety of factors can influence Hg contamination in fish, such as species, place of origin, whether it originates from pisciculture or the natural environment, feed, life span, and size^73^. It is known that fish meal can come from two sources, fishery by-products and fish caught exclusively for the production of this ingredient^76^. Therefore, all of these factors can influence Hg contamination in the fish meal used in pet food. Some studies have shown that fish from pisciculture has less Hg contamination than fish from natural environments^77,78^.

In Brazil, salmon (scientific name: *Salmo salar*) is widely used in fish meal employed by the pet food industry and, according to Ginsberg^79^, regarding the risk of Hg poisoning in humans, salmon is not worrisome. Furthermore, in the study conducted by Olmedo^80^, in which toxic metals were evaluated in several fish species, low Hg concentrations were found in salmon samples, of which the median and interval between 5 and 95th percentiles were 0 (0–0.004). This could explain the Hg concentrations below the detection limit in the fish meal samples analyzed in the present study. In addition, fish meal may come from waste from the tilapia filleting industry^81^, the most produced fish species in Brazil^82^, and low Hg concentrations in this species have been reported. In the study by Kitahara^74^, where samples of 11 different species of freshwater fish were analyzed, tilapia was the one with the lowest concentrations, ranging from 0.01 to 0.02 mg/kg. This could also justify the non-detection of mercury in the fish meal analyzed in our study.

In reference to the Hg contents in the analyzed carbohydrate sources, this toxic metal was not quantified above the detection limit in any whole corn sample, and wheat bran did not differ from broken rice. Among protein sources, in addition to fish meal, no Hg was present above the detection limit in any sample of corn gluten meal 60. Beef meal and chicken by-products meal showed higher concentrations, compared to other sources.

Other ingredients could be considered potential contaminants, such as broken rice since more than 50% of the samples exceeded the MTL more than ten times, as well as corn gluten meal 21, which had values up to 20 times higher than the MTL. These ingredients, in addition to having high Hg concentrations, can be included in high amounts in the formulation of dry foods for dogs and cats and are widely used by the pet food industry. Perhaps this justifies the high Hg concentrations found in the dry foods analyzed in this study. Some samples of swine fat had high Hg concentrations, with a maximum value almost ten times above MTL of Hg, so the inclusion of this ingredient can also significantly influence Hg concentrations in commercial pet food.

### Lead

Regarding Pb concentrations in the analyzed dog and cat foods, 80.55% and 32.14% exceeded the MTL for this element, respectively. Although a large portion of the foods, especially those intended for dogs, exceeded the MTL, only two dog foods and one cat food exceeded the MTL more than two times. Similar results were observed by Duran^40^ in pet foods marketed in Turkey. In contrast, in another study evaluating commercial pet foods sold in Brazil, lower Pb concentrations (0.05–1.4 mg/kg) were observed^61^. Pedrinelli^57^ also observed Pb concentrations above the MTL in homemade foods for dogs and cats prepared with Brazilian ingredients.

Of the analyzed ingredients, wheat bran was the carbohydrate source with the highest Pb levels, while the beef meal was the protein source that presented the highest concentrations of this toxic metal. In addition, values more than two times above the Pb MTL were observed in samples of chicken by-products meal, feather meal, and fish meal, which can contribute to the contamination of the final product if these ingredients are used in high quantities. The higher Pb levels found in beef meal corroborate the results found in the study performed by Kim^44^, higher Pb concentrations were observed in dog foods, in which red meat was the main protein source based on the list of ingredients stated on the label, compared to foods composed mainly of chicken and fish proteins. In that study, the maximum Pb concentration was also found in red meat-based foods, which was 270 times higher than the average human daily intake in units per megacalories estimated by Thomas^83^. The medians of the results found in the chicken- and fish-based foods exceeded the average daily human intake by 6 and 8 times, respectively.

As for the mineral supplements analyzed, high Pb concentrations were observed in the samples of calcium carbonate and dicalcium phosphate, which exceeded the MTL by 6.63 and 9.25 times, respectively. Although these ingredients are used in small quantities, compared to carbohydrate and protein sources, they could also contribute to the contamination of the final product. Environmental contamination by Pb is mainly due to the burning of fossil fuels (coal, natural gas, and oil) and the mining industry. In the past, tetraethyl lead was added to increase the gasoline octane rating, and the burning of this fuel was considered the main source of environmental contamination by Pb. Although this practice was banned in 1989, a large part of the Pb present in Brazilian soils is still attributed to tetraethyl lead^81^, which may justify the results observed in the present study.

With regard to the adverse effects of Pb excess, it is known that its toxicity may involve gastrointestinal signs^50,51^, neurological disorders^50,52^, damage to the hematopoietic system^53,54^, and kidney injuries^52^. Pb MTL was established based on a study in which there were no adverse effects related to the consumption of a diet with 10 mg/kg of Pb in dogs for a period of 2 years^84^. It is not known whether the Pb levels in the foods evaluated in the present study that exceeded the MTL can cause adverse effects, noting that three foods exceeded this limit by more than two times. In the study conducted by Steiss^85^, neuropathy or histological changes in the central nervous system were not observed in dogs that consumed an oral dose of 5 mg of Pb/kg BW/day for 40 weeks. Two dogs, however, showed evidence of non-regenerative anemia after 24 and 26 weeks of consumption. In our study, through the simulation of Pb consumption per kg of BW, the highest values observed were approximately 0.4 mg/kg BW for dogs and 0.5 mg/kg BW for cats, much lower than the dose studied by Steiss^85^, suggesting a safety margin in the analyzed foods. In addition, according to Wismer^86^, the chronic accumulative toxic dose of Pb for dogs is 1.8–2.6 mg/kg BW/day, much higher than the amount estimated in our study.

### Uranium

Of the analyzed dog and cat foods, 85.71% and 95.85% exceeded the MTL for uranium, respectively. In addition to this large portion having exceeded the MTL, the observed values exceeded this limit by up to 14 times. High U concentrations were also found in homemade foods for dogs and cats in the study performed by Pedrinelli^57^, in which 92% of the recipes for dogs and 100% of the recipes for cats exceeded the MTL, with values up to 16 times higher.

As for the analyzed ingredients, wheat bran was the carbohydrate source that showed the highest U concentrations and, among the protein sources, higher U concentrations were found in the beef meal. Regarding the analyzed mineral supplements, high U concentrations were observed in the samples of calcium carbonate (281.24 mg/kg) and dicalcium phosphate (673.24 mg/kg), which corresponds to 28.12 and 67.32 times the MTL of that element, respectively.

In addition to the high concentrations observed in these ingredients, it is worth mentioning that most of the ingredients of animal and vegetable origin had concentrations much higher than U MTL, such as beef meal, of which all samples evaluated exceeded more than 17.5 times the MTL. All samples of fish meal, soybean meal, and wheat bran exceeded the MTL by more than ten times. In the case of chicken by-products meal, all samples exceeded more than 7.5 times the MTL. Due to the widespread use of these ingredients in the manufacture of commercial pet foods, these results may justify the high U concentrations observed in the commercial pet foods analyzed in this study.

Brazil has the fifth largest U reserve in the world, totaling 309,000 tonnes of this element, representing more than 5% of the total in the world^87^. This could justify the high U concentrations observed in the present study, since the exploration of uranium mines may result in contamination of water and soil and, consequently, of the ingredients used by the pet food industry. Furthermore, according to Prado^88^, contamination with uranium in foods occurs mainly as a consequence of its presence in phosphate rocks, from which fertilizers and mineral supplements used in animal feed, such as phosphate, are extracted. Of all the samples of ingredients analyzed, the sample of dicalcium phosphate showed the highest U concentration (623 mg/kg). The U contamination in plant-based products could be attributed to the use of phosphates as soil fertilizers, whereas the contamination of animal by-products could occur due to the consumption of phosphate by animals as a mineral supplement, as well as the consumption of vegetables contaminated by the continuous use of phosphate as a fertilizer.

The U concentrations in the analyzed foods are worrying, as this metal is considered one of the heaviest toxic elements present in the environment and is a precursor to natural radionuclides, therefore it emits alpha and gamma radiation^89^. In the study conducted by these latter authors, the effects of U consumption by growing dogs on bone U deposition were evaluated, as well as on clinical and histopathological changes. In that study, one Beagle consumed a diet containing 20 mg/kg of uranium and three other dogs of the same breed consumed a diet with 100 mg/kg of uranium for 279 days. On the histopathological examination, glomerular degeneration was observed in the three animals that consumed the food with the highest U content. However, no changes were observed in the animal that consumed the diet with 20 mg/kg of uranium. In our study, two cat foods and 12 dog foods had U concentrations above 100 mg/kg, which could imply risks of glomerular injury. However, it is worth noting that in the study by Arruda‐Neto^89^, growing dogs were evaluated, which are perhaps more susceptible to the harmful effects of uranium, in addition to consuming a larger amount of food (consequently more uranium) per kg of metabolic weight, due to the greater energy and nutritional requirements in this stage of life.

It is noteworthy that for the determination of MTL, due to the lack of information in the literature regarding the U toxicity to dogs and cats, the FDA^59^ used the MTL of the most sensitive mammal to this toxic metal, which in this case are rodents, and reduced this value by ten times, as suggested when extrapolating between species. According to Vicente–Vicente^90^, when comparing dogs and rodents with respect to sensitivity to U inhalation, less sensitivity was observed in dogs. This suggests that perhaps dogs are also less sensitive to U intake than rodents, in this case, there would be no need to reduce the U MTL by ten times when extrapolating rodents to dogs. Therefore, a smaller portion of the analyzed foods would be above this limit. Concerning cats, there is no study to date that has investigated the effects of U consumption or inhalation.

### Vanadium

As for V, 75% and 28.57% of dog and cat foods exceeded the MTL for this element, respectively. However, the majority exceeded less than two times that limit. Due to the lack of information regarding the V toxicity in dogs and cats, the MTL proposed by the FDA^59^ was the V MTL for the mammal known to be more sensitive to this element, divided by 10 (safety factor). Therefore, it is possible that dogs and cats are less sensitive and, in this case, a smaller portion of the samples would have exceeded the MTL. No food exceeded the V MTL for the most sensitive mammal (10 mg/kg) in the present study.

The highest toxic metals concentrations, in general, in animal-based ingredients than plant-based ingredients could be attributed to the potential for the accumulation of these elements in the animal organism. According to NRC^60^, some elements can accumulate in animal tissues, such as bone tissue, skeletal muscle, liver, kidneys, and spleen, as well as in animal products such as milk, reaching critical concentrations that may imply adverse effects on the health of human beings who consumes these products. Also according to NRC^60^, this could happen even in situations in which the levels of these minerals in animal feed are below the MTL for the respective species (considered safe). Therefore, it is possible that food animals, such as broilers and beef cattle, consume a diet with plant-based ingredients contaminated with toxic metals in addition to supplements with a large contamination potential, such as dicalcium phosphate, and accumulate these metals in various body tissues, which results in high contamination of their by-products, such as poultry by-products meal and meat meal. The animal tissues mentioned above are part of the composition, in general, of animal-based ingredients used in the formulation of pet foods, which perhaps justifies the greater contamination of these by-products.

## Conclusion

The analyzed foods presented high concentrations of the following elements: Al, Cu, Hg, Pb, U, V, and Zn. In general, animal-based ingredients have a greater potential for contamination than plant-based ingredients. Further studies are needed to assess the effects of chronic ingestion of the elements mentioned above in the quantities found in this study and if under the same circumstances, these toxic metals pose risks to the dogs' and cats’ health. Regarding the concentrations of the elements As, B, Ba, Be, Cd, Co, Sn, Sb, Ni, and Fe, in general, values above the respective MTLs and/or legal limits were not observed, both in commercial foods and in the ingredients analyzed in the present study, so it is not considered that there are intoxication risks with these elements in small animals through long-term consumption of the analyzed foods. It is noteworthy that it is necessary to establish legal limits for all metals with toxic potential.

## Data Availability

The datasets generated during and/or analyzed in the current study are available from the corresponding author on reasonable request.
